# Self-disproportionation of enantiomers of thalidomide and its fluorinated analogue *via* gravity-driven achiral chromatography: mechanistic rationale and implications†
†Electronic supplementary information (ESI) available. CCDC 1009504, 1009505, 1009506, 1009507, 1009508, 1009509. For ESI and crystallographic data in CIF or other electronic format see DOI: 10.1039/c4sc03047h



**DOI:** 10.1039/c4sc03047h

**Published:** 2014-10-30

**Authors:** Mayaka Maeno, Etsuko Tokunaga, Takeshi Yamamoto, Toshiya Suzuki, Yoshiyuki Ogino, Emi Ito, Motoo Shiro, Toru Asahi, Norio Shibata

**Affiliations:** a Department of Nanopharmaceutical Sciences and Department of Frontier Materials , Nagoya Institute of Technology , Gokiso, Showa-ku , Nagoya 466-8555 , Japan . Email: nozshiba@nitech.ac.jp ; Fax: +81-52-735-7543; b Department of Life Science and Medical Bioscience , Waseda University (TWIns) , Wakamatsu-cho 2-2, Shinjuku-ku , Tokyo 162-8480 , Japan . Email: tasahi@waseda.jp; c Consolidated Research Institute for Advanced Science and Medical Care , Waseda University (ASMeW) , Waseda-tsurumaki-cho 513, Shinjuku-ku , Tokyo 162-0041 , Japan

## Abstract

We report on the self-disproportionation of enantiomers (SDE) of non-racemic thalidomide (**1**) and 3′-fluorothalidomide (**2**) under the conditions of gravity-driven achiral silica-gel chromatography. The presence of a fluorine atom on the chiral center dramatically alters the structure and polarity of **1** and **2**, resulting in the opposite SDE profile on silica-gel.

## Abstract 2

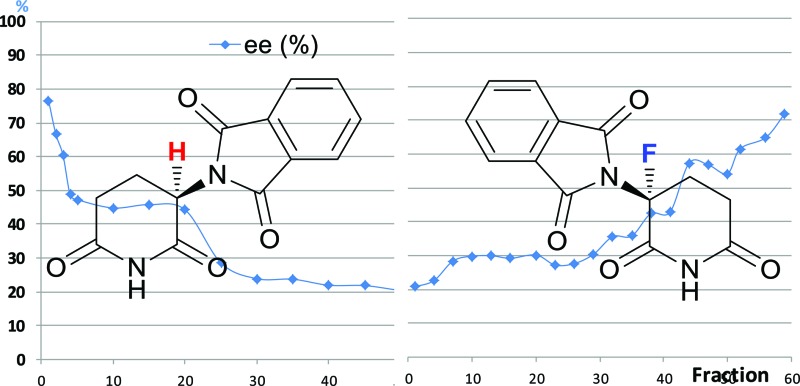
We report on the self-disproportionation of enantiomers of non-racemic thalidomide (**1**) and 3′-fluorothalidomide (**2**) under achiral chromatography.

## 

Thalidomide (**1**) is one of the most notorious drugs in pharmaceutical history due to the humanitarian disaster in the 1950s.^1^ Thalidomide (**1**) possesses a single stereogenic carbon in the glutarimide ring, and it is conceivable that the unexpected teratogenic side effects are ascribed to the (*S*)-enantiomer of **1**.^2^ However, this has been a matter of debate because considerable chiral inversion should take place during the incubation of enantiomerically pure **1**.^3^ Despite the tragic disaster, the unique biological properties of **1** prompted its return to the market in the 21^st^ century for the treatment of multiple myeloma and leprosy.^4^ Furthermore, a large number of papers on novel medical uses of **1** are continuing to appear in the biological and medicinal literature.^4^ We envisage that many kinds of newly discovered biological actions for **1** would account for the concealed physical and chemical properties of **1**, including its chirality.^5^ As one may expect, the physicochemical and chiroptical properties of **1** have been scrupulously studied. However, properties such as the self-disproportionation of enantiomers (SDE)^6^ of **1** has never been studied, despite the fact that it may have direct relation to its physiological behavior.

Self-disproportionation of enantiomers (SDE) was coined by Soloshonok in 2006 (ref. 6) to describe a process by which enantiomerically enriched compounds are separated into fractions of a different proportion of enantiomers (enantiomerically enriched and depleted), compared to the original sample, without the assistance of any external chiral sources.^7^ This phenomenon is fundamentally general and can be expected for any chiral compound being subjected to achiral chromatography,^8^ sublimation^9^ or distillation.^10^ While the phenomenon itself might not be surprising the SDE phenomenon has never been systematically studied and therefore is still unpredictable in terms of the relationship between the observed magnitude of SDE and compound structures.^11^

During our research on thalidomide and its derivatives,^12^ we came across the unique behavior of non-racemic **1** and fluorinated analogue **2** under the conditions of a commonly used gravity-driven achiral chromatography. In this paper, we disclose that both non-racemic **1** and **2** show high magnitude of SDE, but their SDE profiles are completely opposite. Thus, achiral chromatography of non-racemic **1** (35.5% ee) resulted in isolation of enantiomerically enriched **1** (87% ee) in the first fraction while enantiomerically depleted **1** (21% ee) was observed in the last fraction. On the other hand, **2** with a highest ee of 71%, was eluted in the last fraction under similar achiral chromatographic conditions, while **2** with at lowest 30% ee was found in the first fraction, different from the original ee of **2**, 34%. X-ray crystallographic analysis and computations of **1** and **2** revealed that the introduction of a single fluorine atom in the chiral center of **1** dramatically altered the monomeric and dimeric structures, and log *P* values of **1**. The opposite behaviors of **1** and **2** on SDE can be explained by the difference of aggregations and polarities of chiral, non-racemic **1** and **2** and racemic **1** and **2** (Fig. 1).

**Fig. 1:**
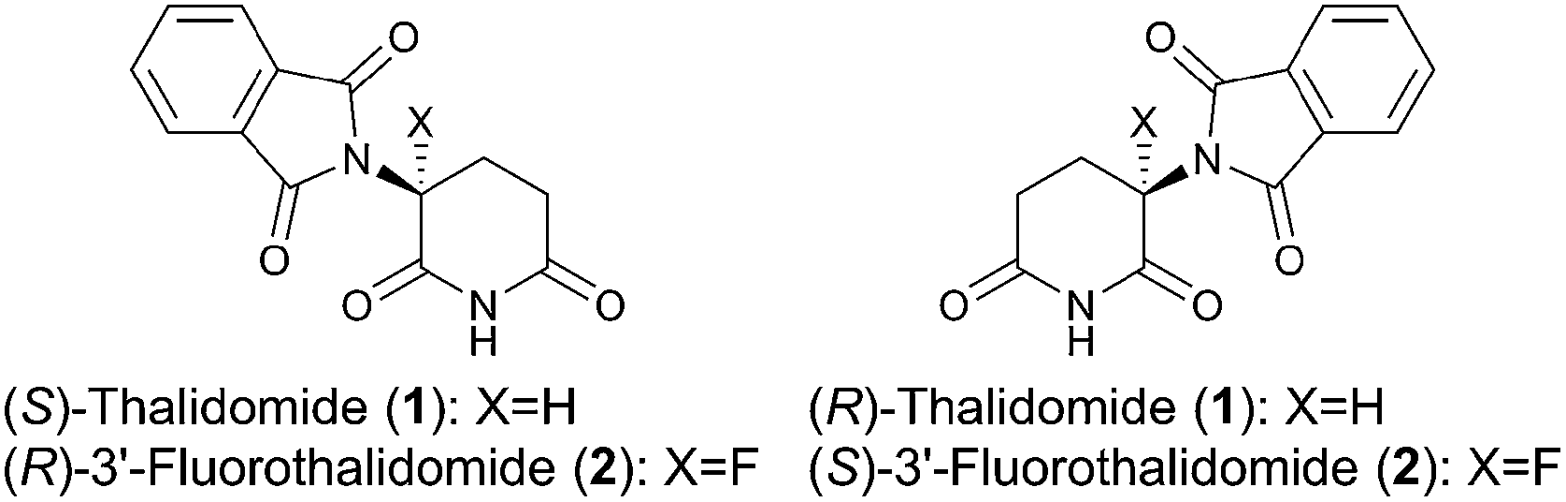
Thalidomide (**1**) and 3′-fluorothalidomide (**2**).

An experiment was conducted to examine whether SDE occurs for **1** under conventional chromatographic conditions with regular silica-gel on an achiral stationary phase. Partially enantioenriched (*R*)-**1** (*ca.* 40% ee) served as the loading substrate. Table 1 shows the data for the experiment involving the SDE of **1** during achiral silica-gel chromatography. We first attempted to separate **1** on a glass column of 10 mm diameter and 50 mm length filled with regular silica-gel (KANTO CHEMICAL CO., INC., Silica Gel 60N, spherical, neutral, 63–210 μm) as the stationary phase at atmospheric pressure using DMSO as the loading solvent. The difference between minimum and maximum ees for the chromatographic fractions is shown as an evaluation value of this phenomenon. The SDE was not observed under dichloromethane/methanol (95/5) eluent (run 1). SDE was pronounced when hexane/ethyl acetate (5/5) was used as the eluent, and good Δee was obtained using hexane/ethyl acetate (7/3) (runs 2 and 3). The use of DMF or dioxane for loading decreased the Δee values (runs 4 and 5).

**Table 1:** Initial experiments of SDE of (*R*)-**1** (41.6% ee) during achiral silica-gel chromatography

Run^*a*^	Loading solvent	Eluent	% ee min^*b*^	% ee max^*b*^	Δee^*c*^
1	DMSO	DCM/MeOH = 95/5	—	—	—
2	DMSO	H/A = 5/5	21.2	57.8	36.6
3	DMSO	H/A = 7/3	20.3	71.3	51.0
4	DMF	H/A = 7/3	25.6	66.5	40.9
5	Dioxane	H/A = 7/3	26.2	64.9	38.7

^*a*^Regular silica-gel packed in a glass column (10 × 50 mm) was used under atmospheric pressure.

^*b*^ee was determined by HPLC using a CHIRALCEL OJ-H with ethanol as the eluate.

^*c*^Δee = (% ee max) – (% ee min).

Next, separation was attempted using various silica-gels as column packing materials with different column lengths under hexane/ethyl acetate (7/3) conditions (Table 2). The Δee observed by flash silica-gel (KANTO CHEMICAL CO., INC., Silica Gel 60N, spherical, neutral, 40–50 μm) was better than that by regular silica-gel pre-treated with water (10 wt%) (runs 1 and 2). The highest Δee for **1** was 66.2% on a column filled with mesoporous silica-gel (run 3). The Δee improved on a longer column (runs 4 and 5). It should be noted that the phenomenon of SDE is quite general for **1** under ubiquitous purification conditions such as silica-gel/ethyl acetate–hexane. When we attempted separation of **1** using Al_2_O_3_, the SDE effect was not significant and a fluctuating performance was observed (run 6).

**Table 2:** Optimization of the self-disproportionation of enantiomers of (*R*)-**1** during achiral silica-gel chromatography

Run^*a*^	Starting ee of (*R*)-**1** (%)	Silica-gel	% ee min^*b*^	% ee max^*b*^	Δee^*c*^
1	35.5	Regular^*d*^	27.4	80.9	53.5
2	41.6	Flash	23.8	83.1	59.2
3	35.5	Mesoporous	20.7	86.9	66.2
4^*e*^	41.6	Flash	17.7	80.1	62.4
5^*f*^	36.2	Flash	15.0	80.0	65.0
6	31.1	Al_2_O_3_	21.6	34.5	12.9

^*a*^Achiral silica-gel packed in a glass column (10 × 50 mm) was used under atmospheric pressure. DMSO was used as the solvent for loading.

^*b*^ee was determined by HPLC using a CHIRALCEL OJ-H with ethanol as the eluate.

^*c*^Δee = (% ee max) – (% ee min).

^*d*^Silica-gel was wetted with 10 wt% water.

^*e*^A 10 × 80 mm column was used.

^*f*^A 10 × 110 mm column was used.

SDE was also observed for the 3′-fluorinated analogue of thalidomide, **2** (Table 3). When we attempted to separate **2** using a glass column filled with regular silica-gel, the SDE effect was not significant and **2** was partially decomposed during purification (run 1). When mesoporous silica-gel was used instead, a low Δee value was obtained without decomposition of **2** (run 2). Separation was next performed on silica-gel pre-treated with water to prevent decomposition. Although the SDE effect was unsuccessful using regular silica-gel pre-treated with 5 wt% water (run 3), a moderate Δee value was obtained on regular silica-gel with 10 wt% water or flash silica-gels with 5 and 10 wt% water (runs 4–6). When we attempted separation of **2** using Al_2_O_3_, the SDE effect was not significant and a fluctuating performance was again observed (run 7).

**Table 3:** SDE of (*R*)-**2** during achiral silica-gel chromatography

Run^*a*^	Starting ee of **2** (%)	Silica-gel	% ee min^*b*^	% ee max^*b*^	Δee^*c*^
1	25.0	Regular	14.7	26.7	12.0
2	34.2	Mesoporous	32.2	56.1	23.9
3	34.2	Regular^*d*^	31.9	38.6	6.7
4	34.2	Regular^*e*^	30.0	70.6	40.6
5	27.3	Flash^*d*^	14.4	52.5	38.1
6	27.3	Flash^*e*^	9.4	50.4	41.0
7	37.2	Al_2_O_3_	27.4	38.6	11.2

^*a*^Achiral silica-gel packed in a glass column (10 × 50 mm) was used under atmospheric pressure. DMSO was used as the solvent for loading.

^*b*^ee was determined by HPLC using a CHIRALCEL OJ-H with ethanol as the eluate.

^*c*^Δee = (% ee max) – (% ee min).

^*d*^Silica-gel was wetted with 5 wt% water.

^*e*^Silica-gel was wetted with 10 wt% water.

With these results in hand, we investigated the relationship between the ee value and mass of each fraction, which was estimated based on the peak area of **1** and **2** after HPLC analysis since the total recoveries of **1** and **2** were quantitative at the end of the chromatographic separation for each experiment. Fig. 2 shows the details of chromatography of **1** with an ee value of 36.3% using a 10 × 50 mm column filled with flash silica-gel (Fig. 2a), and **2** (32.0% ee) using a 10 × 50 mm column filled with regular silica-gel over wetted 10 wt% water (Fig. 2b). In the case of **1**, the first fraction has the highest ee value and the ee values decreased gradually as the fractions increased. The last ee value converged to a lower ee than that of the loading sample. On the other hand, in the case of fluorinated **2**, the first fraction had the lowest ee value and the ee values increased as the fraction number increased. The highest ee of **2** was observed in the last fraction. The masses are described by a parabola-like curve in both cases.

**Fig. 2:**
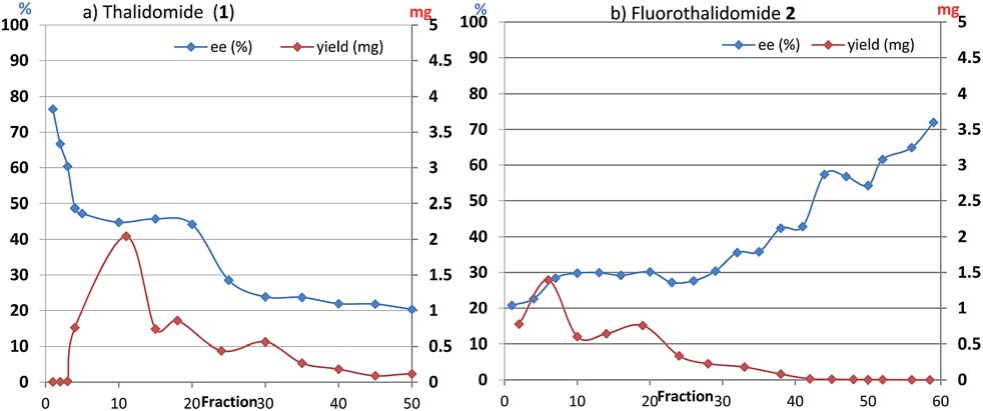
(a) Ees and yields with fraction numbers during the separation of (*R*)-**1** (36.3% ee) on a column (10 × 50 mm) filled with mesoporous silica-gel. (b) Ees and yields with fraction numbers during the separation of (*R*)-**2** (32.0% ee) on a column (10 × 50 mm) filled with regular silica-gel wetted 10 wt% water.

The basic mechanism for the phenomenon of SDE has been proposed to involve homochiral *vs.* heterochiral high-order species with different molecular weights such as monomers, dimers or oligomers, allowing their separation under the condition of achiral chromatography.^8^ We therefore considered the potential formation of heterochiral or homochiral dimers in the intermolecular interactions between the enantiomers of **1** in solution leading to the manifestation of the SDE. In our previous report of the X-ray crystal structure analysis of **1**, the racemic mixture, (*R*/*S*)-**1** forms symmetrical (*R*/*S*)-heterochiral dimers, and (*S*)-**1** is found as asymmetrical (*S*/*S*)-homochiral dimers in the crystals.^13^ The crystals were taken from MeOH–water. The X-ray crystal structures show the differences in the hydrogen-bonded lengths between heterochiral and homochiral dimers. The hydrogen bonds in (*R*/*S*)-heterochiral dimers are slightly shorter than those of asymmetrical (*S*/*S*)-homochiral dimers (Fig. 3). In addition, the heterochiral dimer was estimated to be approximately 1 kcal mol^–1^ more stable than the homochiral dimer by theoretical calculations.^13^

**Fig. 3:**
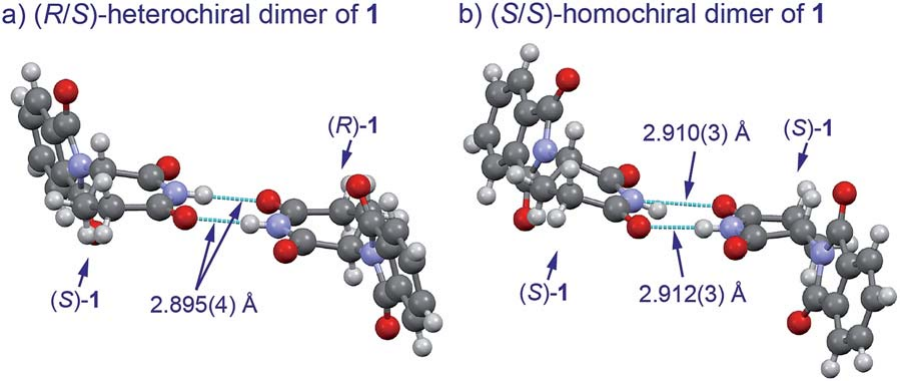
X-Ray crystallographic structures of racemic **1** (monoclinic, ESI†) and (*S*)-**1** (monoclinic, ESI†).^10^

An understanding of the conformational changes and aggregation states of thalidomide by fluorine-replacement leads to additional insight into the mechanisms of SDE. X-Ray crystal structures of racemic **2**, and (*S*)-**2** were next investigated (crystallized from ethanol). To our great astonishment, both structures of **2** were very different from the parent, non-fluorinated thalidomide (**1**) despite their sterically isosteric relationship. While racemic **2** shows the structure of a (*R*/*S*)-heterochiral dimer, (*S*)-**2** exists as a monomer without any hydrogen bonding between enantiomers. Even more interestingly, in racemic **2**, the hydrogen bonding system between (*R*)-**2** and (*S*)-**2** is entirely different from that of the (*R*/*S*)-heterochiral dimer of racemic **1** (Fig. 4). These significant differences are likely to be attributed to a significant conformational change, as compared to original thalidomide, induced by the presence of the fluorine atom. In thalidomide (**1**), a sterically demanding phthalimido group occupies the equatorial position. On the other hand, fluorine is located at the equator and the phthalimido moiety at the axial position, despite its steric bulkiness (Fig. 5). Although the reason for the fluorine effect on the conformational change is not clear, it could be explained by the electrostatic repulsion between the fluorine and the two carbonyls of the piperidine-2,6-dione ring, and/or a strong dipole induced by the fluorine atom.^14^ Namely, the equatorial-fluorine conformation of **2** is presumably preferable, since the fluorine exists on the same plane as the two carbonyls of piperidine-2,6-dione in **2**. On the other hand, the fluorine is almost perpendicular to the two carbonyls in the axial-fluorine conformation of **2**, resulting in less-stabilization. The computations (DFT, B3LYP/6-311+G(d,p)) also support these results that the phthalimide moiety of **1** occupies an equatorial place while the fluorine occupies the equatorial position in **2** (Fig. 6).

**Fig. 4:**
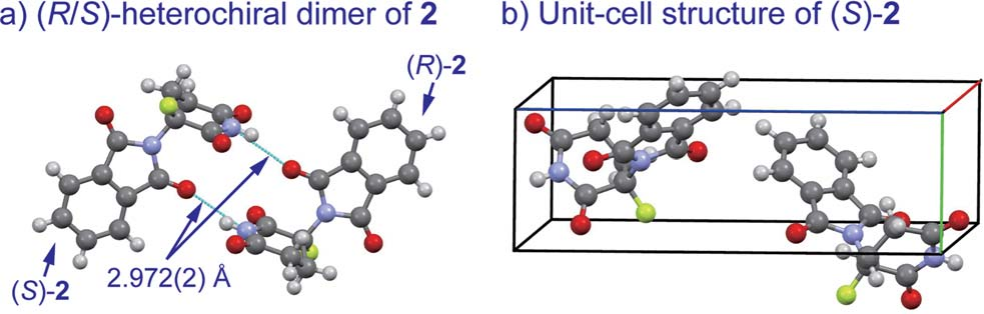
X-Ray crystallographic structures of racemic **2** (monoclinic, ESI†) and (*S*)-**2** (monoclinic, ESI†).

**Fig. 5:**
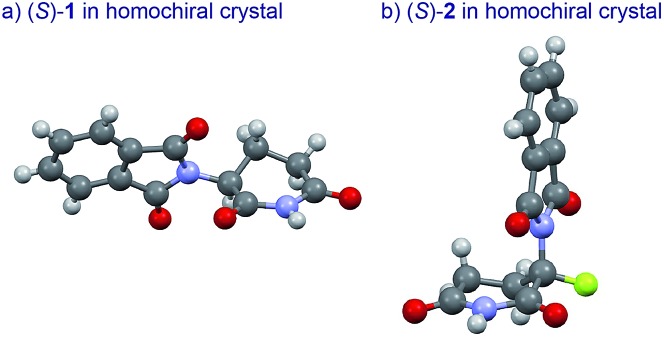
X-Ray crystallographic structures of (*S*)-**1** (ESI†) and (*S*)-**2** (ESI†).

**Fig. 6:**
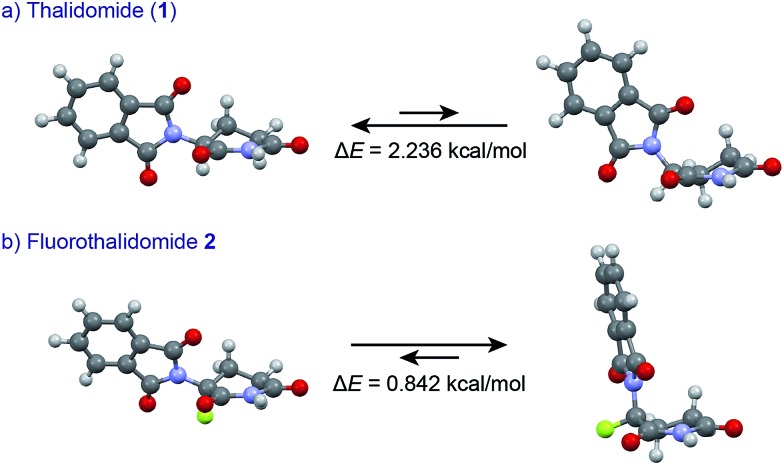
Comparisons of conformational stability of **1** and **2** by DFT calculations (B3LYP/6-311+G(d,p)).

Crystal structures of racemic thalidomide have been investigated since 1971.^5*c*^ The existence of polymorphism of racemic **1** has been suggested with the relationship between its different physical forms and dissolution behavior.^5d,5h,5i^ We thus re-attempted to grow crystals of fluorinated thalidomide **2** using different solvents. In this attempt, (*R*)-**2** was taken from chloroform and acetonitrile solutions. Similar to the case of (*S*)-**2**, *i.e.*, crystals from ethanol (Fig. 4b), the unsolvated monomeric structures were revealed without detecting dimerization structures from both chloroform and acetonitrile solutions. Interestingly, while crystals of (*R*)-**2** (α-form, monoclinic, Fig. 7a, ESI†) obtained from acetonitrile are the same as (*S*)-**2** from ethanol (Fig. 4b), an alternate arrangement of (*R*)-**2** was obtained from chloroform solution (β-form, orthorhombic, Fig. 7b, ESI†), with an infinite hydrogen bonded chain in (*R*)-**2** (Fig. 7b). It should be mentioned that optically pure **2** is always obtained as a “monomer” independent of crystal solvents, while all attempts for the crystallization of racemic **2** gave the same crystal system of monoclinic.

**Fig. 7:**
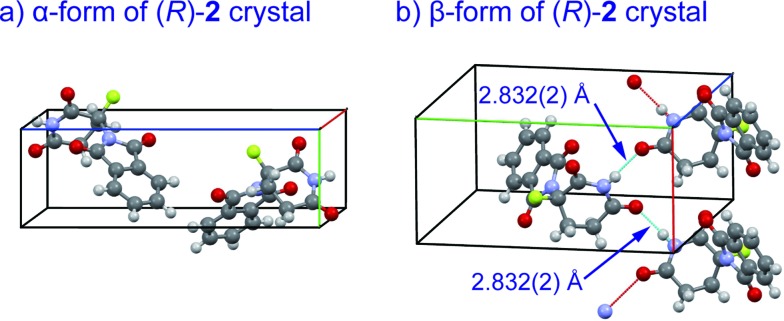
X-Ray crystallographic structures of unsolvated crystals of (*R*)-**2**; (a) α-form (monoclinic, ESI†); (b) β-form (orthorhombic, ESI†) with infinite hydrogen bonded chain.

Starting with the X-ray crystallographic structures, we further estimated the log *P* values of (*R*)-**1**, (*R*/*S*)-heterochiral dimer **1**, (*R*)-**2**, and (*R*/*S*)-heterochiral dimer **2** using DFT computations (B3LYP/6-31+G(d,p)) to be able to discuss the SDE on achiral silica-gel, since the holding time of substrates during silica-gel column chromatography are likely to be intricately related with the polarity of the substrates. The calculated log *P* values are: –0.15 for (*R*)-**1**; –0.30 for (*R*/*S*)-heterochiral dimer **1**; 0.53 for (*R*)-**2**; 1.07 for (*R*/*S*)-heterochiral dimer **2**. The computations indicated that thalidomide (**1**) changes to become more hydrophilic by the formation of its dimer, while fluorinated thalidomide (**2**) becomes more hydrophobic with dimerization.

Structural differences, aggregation states, and log *P* values of **1**, **2** and their enantiomers suggest the supposed mechanisms of SDE of **1** and **2**. Enantioenriched (*R*)-**1** exists as a mixture of (*R*)-enantiomer **1** and racemate **1**. Both (*R*)-enantiomer **1** and racemic **1** form dimers. However, the (*R*/*R*)-homochiral dimer from (*R*)-**1** is less stable than the (*R*/*S*)-heterochiral dimer from racemic **1** based on the calculations. (*R*)-Enantiomer **1** becomes a monomer on silica-gel during elution while racemic **1** tends to stay as a dimer. Hence, enantioenriched (*R*)-**1** was eluted first as a monomer while racemic **1** was eluted in the last fraction as a dimer, due to the difference in log *P* values ((*R*)-**1**: –0.15; (*R*/*S*)-heterochiral dimer **1**: –0.30). In the case of fluorinated thalidomide (**2**), (*R*)-**2** exists as a monomer independent of solvent while racemic **2** forms a dimer. The log *P* values of the monomer and dimer show an opposite tendency to non-fluorinated thalidomide ((*R*)-**2**: 0.53 *vs.* (*R*/*S*)-heterochiral dimer **2**: 1.07). Consequently, racemic **2** (a dimer form) was observed in the first fraction while (*R*)-**2** (a monomer form) was observed in the final fraction (Fig. 8).

**Fig. 8:**
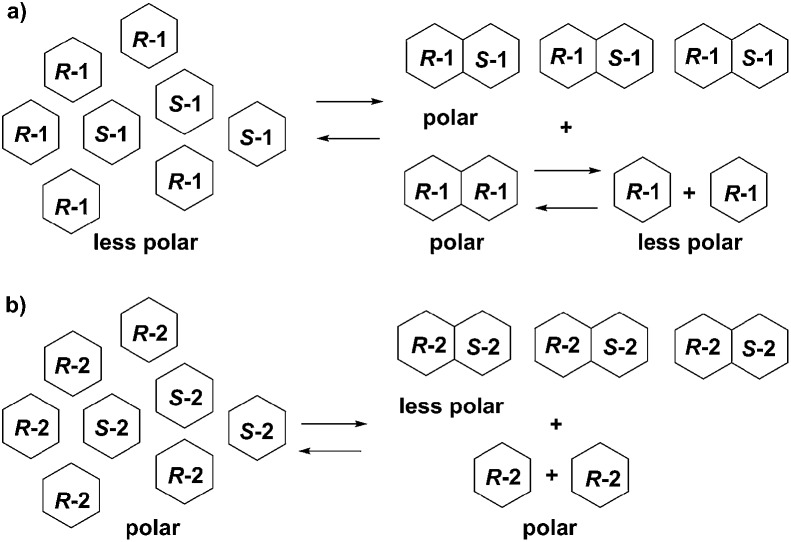
Proposed mechanisms for the opposite behaviors of SDE of (a) enantioenriched **1** and (b) enantioenriched **2**.

## Conclusions

In conclusion, we discovered that thalidomide (**1**) and its fluorinated analogue **2** have a very strong magnitude of self-disproportionation of enantiomers under the conditions of achiral gravity-driven silica-gel chromatography. Remarkably, sterically very similar compounds **1** and **2** were found to have opposite orders of elution of enantiomerically enriched and depleted fractions. Whereas the first fractions of **1** had the highest ee value, chromatography of **2** gave the most enantiomerically enriched samples in the last fractions. Unprecedentedly, simple replacement of single hydrogen by fluorine on the asymmetric carbon dramatically changes the properties of parent molecules including X-ray crystallographic structures, aggregation patterns and polarities which result in the unique, opposite SDE profiles. The results obtained have two major implications: first, the SDE can be used as a nonconventional enantiomer purification method for the preparation of enantiomerically pure samples of thalidomide and its analogs for proper biological/medicinal studies. Second: the discovered SDE profile for thalidomide can have a role in the manifestation of its biological properties. Thus, the teratogenic activity of thalidomide can be attributed not to its single enantiomer but to the heterochiral dimer, a strong preference for which was discovered in this SDE study. This possibility was rather overlooked in the previous studies and we are currently working towards this direction.^15^
