# Supplementary material for: Self-disproportionation of enantiomers of thalidomide and its fluorinated analogue via gravity-driven achiral chromatography: mechanistic rationale and implications
Source: Chem Sci. 2014 Oct 30;6(2):1043–8. doi: 10.1039/c4sc03047h (PMC5811091; doi:10.1039/c4sc03047h)

Electronic Supplementary Material (ESI) for Chemical Science.
This journal is © The Royal Society of Chemistry 2014

SUPPORTING INFORMATION

Self-disproportionation of Enantiomers of Thalidomide and its Fluorinated Analogue via Gravity-driven Achiral Chromatography: Mechanistic Rationale and Implications

Mayaka Maeno, Etsuko Tokunaga, Takeshi Yamamoto, Toshiya Suzuki, Yoshiyuki Ogino, Emi Ito, Motoo Shiro, Toru Asahi* and Norio Shibata*

General information

Silica-gel chromatographies were monitored by thin-layer chromatography (TLC) carried out on 0.25 mm Merck silica-gel (60-F254). The TLC plates were visualized with UV light (302 nm). The ^{1}H-NMR (300 MHz), ^{19}F-NMR (282 MHz) spectra for solution in $CDCl_3$ were recorded on a Varian Mercury 300. Chemical shifts (δ) are expressed in ppm downfield from TMS or $CFCl_3$. Mass spectra were recorded on a SHIMADZU LCMS-2010EV. HPLC analyses were performed on a JASCO U-2080 Plus using 4.6 x 250 mm CHIRALCEL OJ-H column. Infrared spectra were recorded on a JASCO FT/IR-200 spectrometer. (±)-**1**[1], (±)-**2**[2a, 2b], (*R*)-**1**[3], and (*R*)-**2**[4] were prepared according to previously reported procedures. Their enantiomeric mixtures were prepared using (±)-**1** 100 mg and (*R*)-**1** 50 mg or (±)-**2** 100 mg and (*R*)-**2** 50 mg.

(±)-Thalidomide (1)

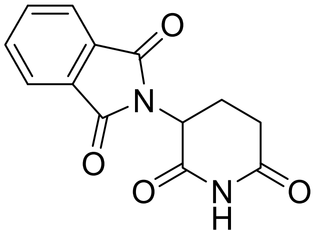

A white solid. ^{1}H NMR (300 MHz, $CDCl_3$): δ 7.97 (bs, 1H), 7.91-7.87 (m, 2H), 7.80-7.76 (m, 2H), 5.00 (dd, *J* = 12.2, 5.1 MHz, 1H), 2.95-2.75 (m, 3H), 2.19-2.14 (m, 1H); IR (KBr): 3195, 3098, 1772, 1710, 1387, 1327, 1259, 1209, 1114, 1091, 1019, 1001, 890, 859, 7278, 607 cm^{-1}; MS (ESI, m/z) calculated for $C_{13}H_{10}N_2NaO_4$ ([M + Na]+) 281.05, found 280.90

(*R*)-Thalidomide (**1**)

A white solid. Spectral data for (*R*)-**1** (^{1}H NMR, IR, MS) corresponded to (±)-**1**. HPLC (DAICEL CHIRALCEL OJ-H, 4.6×250 mm, EtOH=100, flow rate 0.5 mL/min, λ=254 nm) t_R = 12.75 min (major).

(±)-3'-Fluorothalidomide (2)

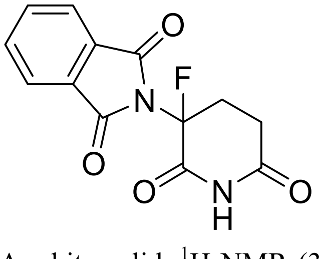

A white solid. ^{1}H NMR (300 MHz, $CDCl_3$): δ 8.07 (bs, 1H), 7.96-7.90 (m, 2H), 7.87-7.83 (m, 2H), 3.64-3.56 (m, 1H), 2.93-2.86 (m, 1H), 2.67-2.48 (m, 2H); ^{19}F NMR (282 MHz, $CDCl_3$): δ −131.51 (s, 1F); IR (KBr): 3317, 3175, 3100, 1798, 1738, 1699, 1365, 1331, 1205, 1117, 1042, 873, 837, 715, cm^{-1}; MS (ESI, m/z) calculated for $C_{13}H_9FN_2NaO_4$ ($[M + Na]^+$) 299.04, found 298.95

(*R*)-3'-Fluorothalidomide (2)

A white solid. Spectral data for (*R*)-**2** (^{1}H NMR, ^{19}F NMR, IR, MS) corresponded to (±)-**2**. HPLC (DAICEL CHIRALCEL OJ-H, 4.6×250 mm, EtOH=100, flow rate 0.5 mL/min, λ=254 nm) t_R = 12.49 min (major).

Typical purification experiment using a column chromatography with an achiral phase

3 g of silica-gel (60N spherical neutral size 63-210 µm or 40-50µm) was packed in a 10 mm x 50 mm glass column with hexane and ethyl acetate as the eluent under atmospheric pressure at room temperature. In general, a solution of 10.0 mg of **1** or **2** dissolved in 0.15 mL of DMSO was loaded on this packed column following which this column was pressurized at the abovementioned pressure and 50-60 (each 2.0 mL) fractions were collected until no more **1** or **2** were detected by TLC analysis. Each fraction was then subjected to high-performance liquid chromatography (HPLC) analysis to determine enantiomeric excess (ee).

References

[1] G. W. Muller, W. E. Konnecke, A. M. Smith, V. D. Khetani, *Org. Process Res. Dev.* **1999**, *3*, 139-140.

[2] a) Y. Takeuchi, T. Shiragami, K. Kimura, E. Suzuki, N. Shibata, *Org. Lett.* **1999**, *1*, 1571–1573; b) selectfluor® (1.5 equiv.) was used instead of $FClO_3$.

[3] E. Suzuki, N. Shibata, *Enantiomer* **2001**, *6*, 275-279.

[4] T. Yamamoto, Y. Suzuki, E. Ito, E. Tokunaga, N. Shibata, *Org. Lett.* **2011**, *13*, 470-473.

HPLC chromatograms of (±)-**1** and (*R*)-**1**

DAICEL CHIRALCEL OJ-H, 4.6×250 mm, EtOH=100, flow rate 0.5 ml/min, λ=254 nm

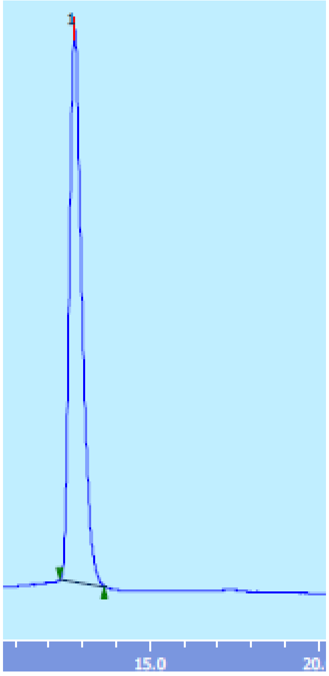

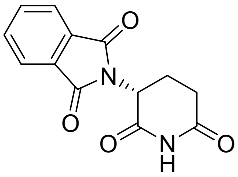

(*R*)-**1**: 99% ee

PK No	Time	Area%
1	12.750	100

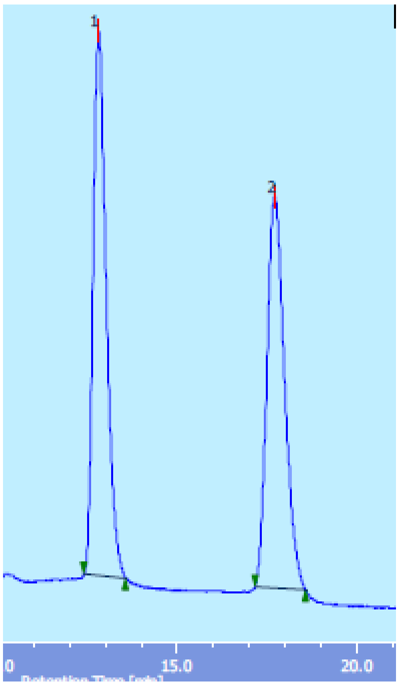

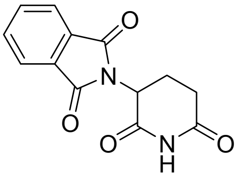

(±)-**1**

PK No	Time	Area%
1	12.767	50.762
2	17.675	49.238

HPLC chromatograms of (±)-**2** and (*R*)-**2**

DAICEL CHIRALCEL OJ-H, 4.6×250 mm, EtOH=100, flow rate 0.5 ml/min, λ=254 nm

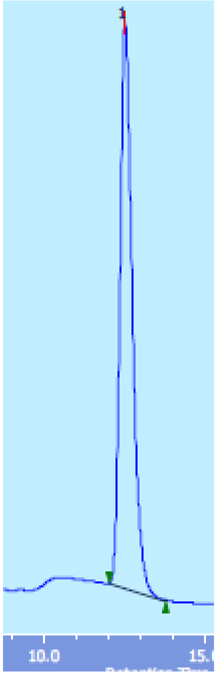

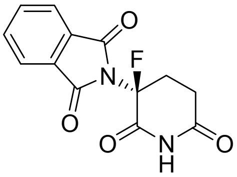

(*R*)-**2**: 99% ee

PK No	Time	Area%
1	12.492	100

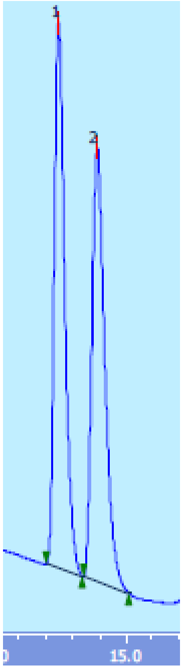

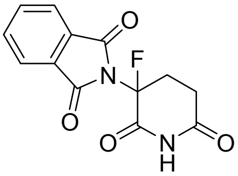

(±)-**2**

PK No	Time	Area%
1	12.475	50.719
2	13.908	49.281

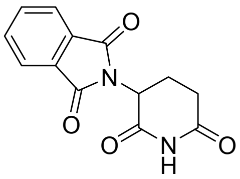

(±)-**1**

^{1}H NMR $CDCl_3$

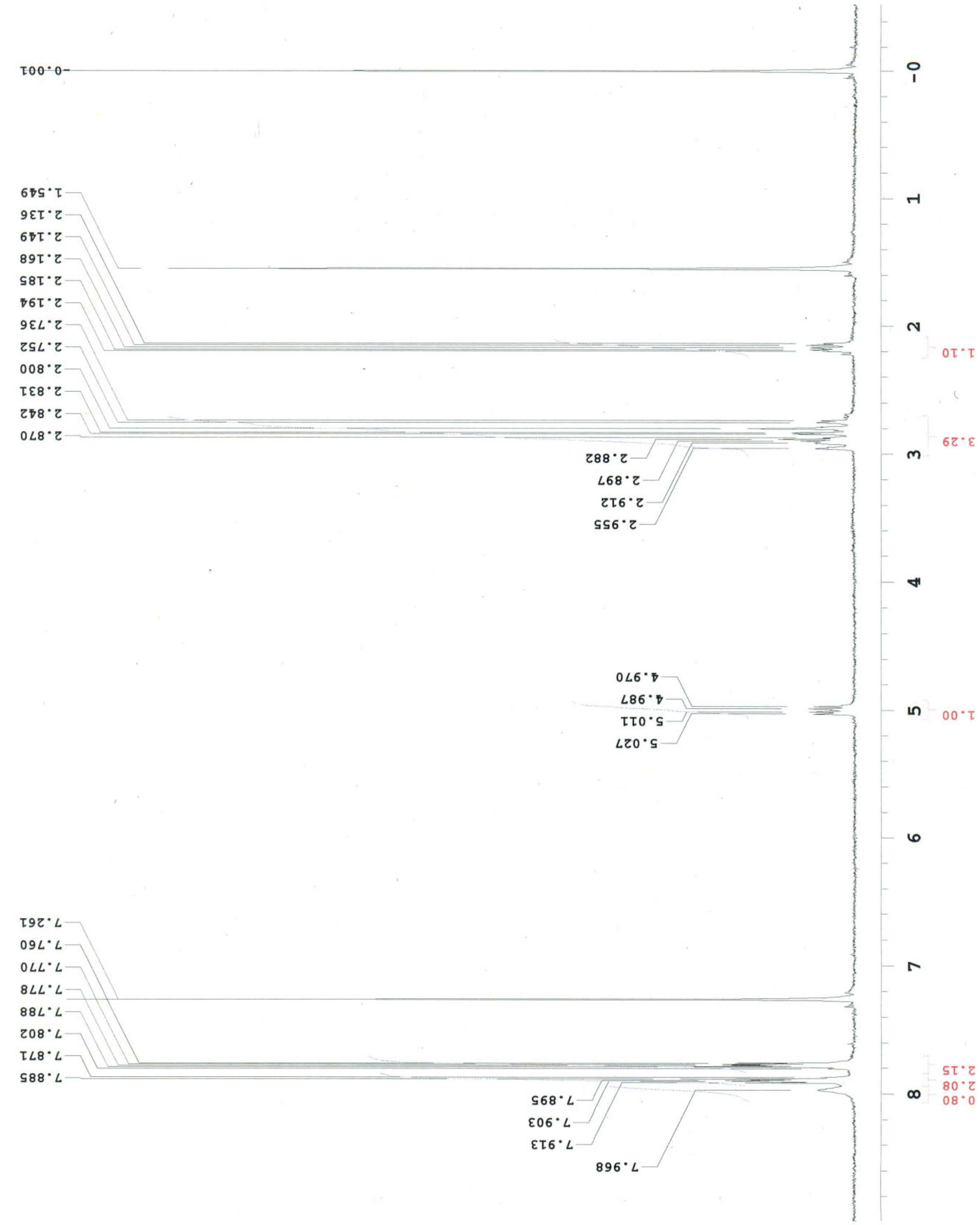

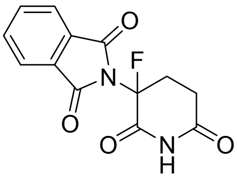

(±)-**2**

^{1}H NMR $CDCl_3$

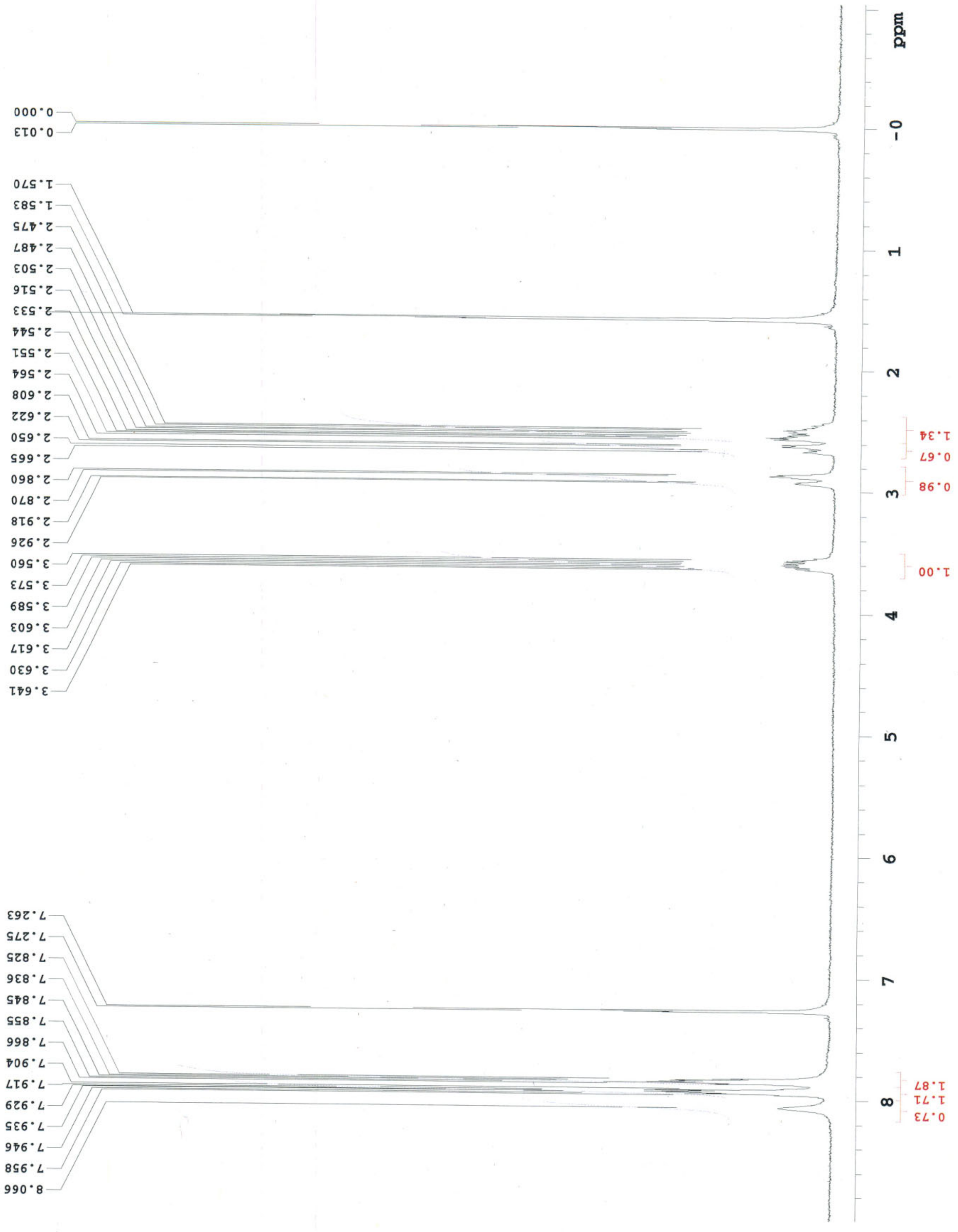

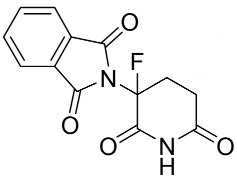

(±)-**2**
^{19}F NMR $CDCl_3$

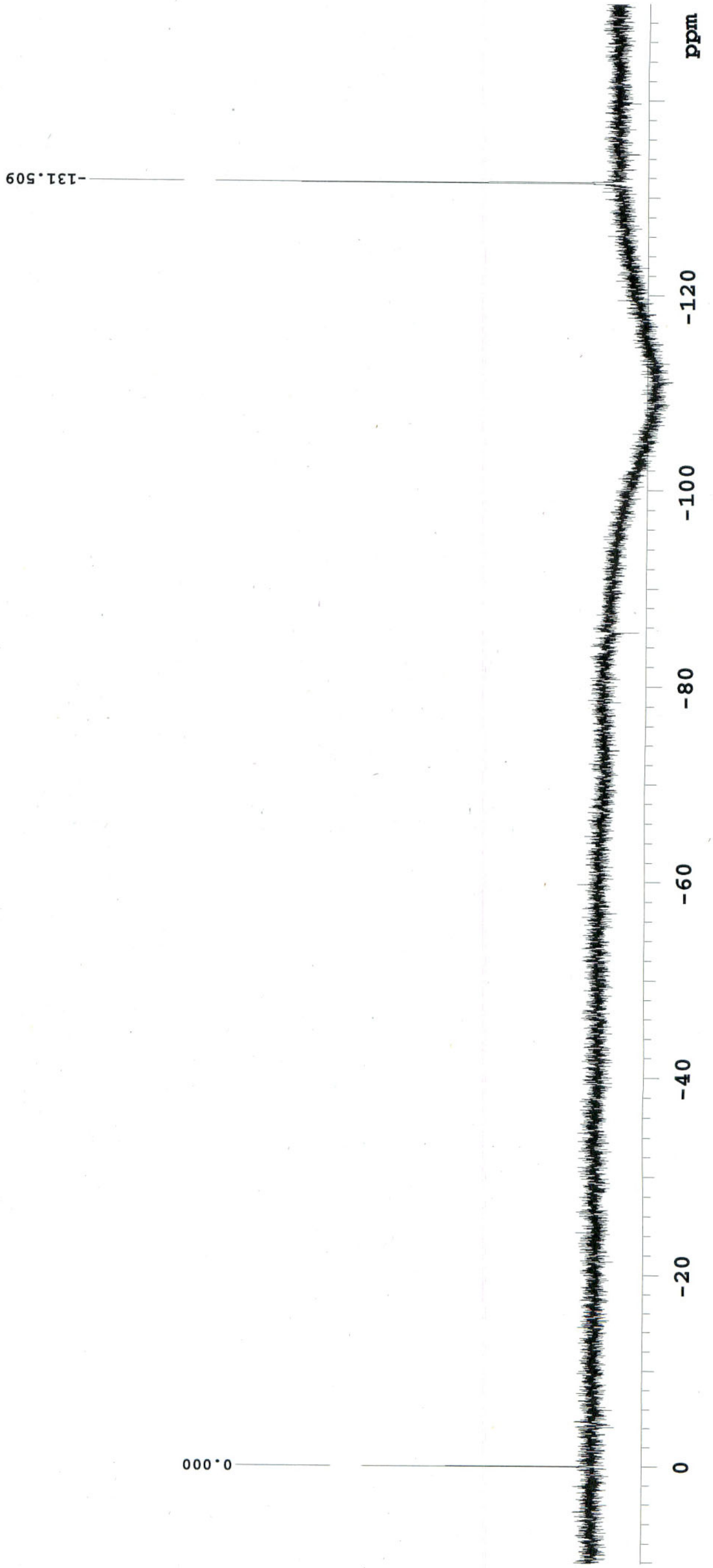

IR (KBr) spectra of (±)-**1**

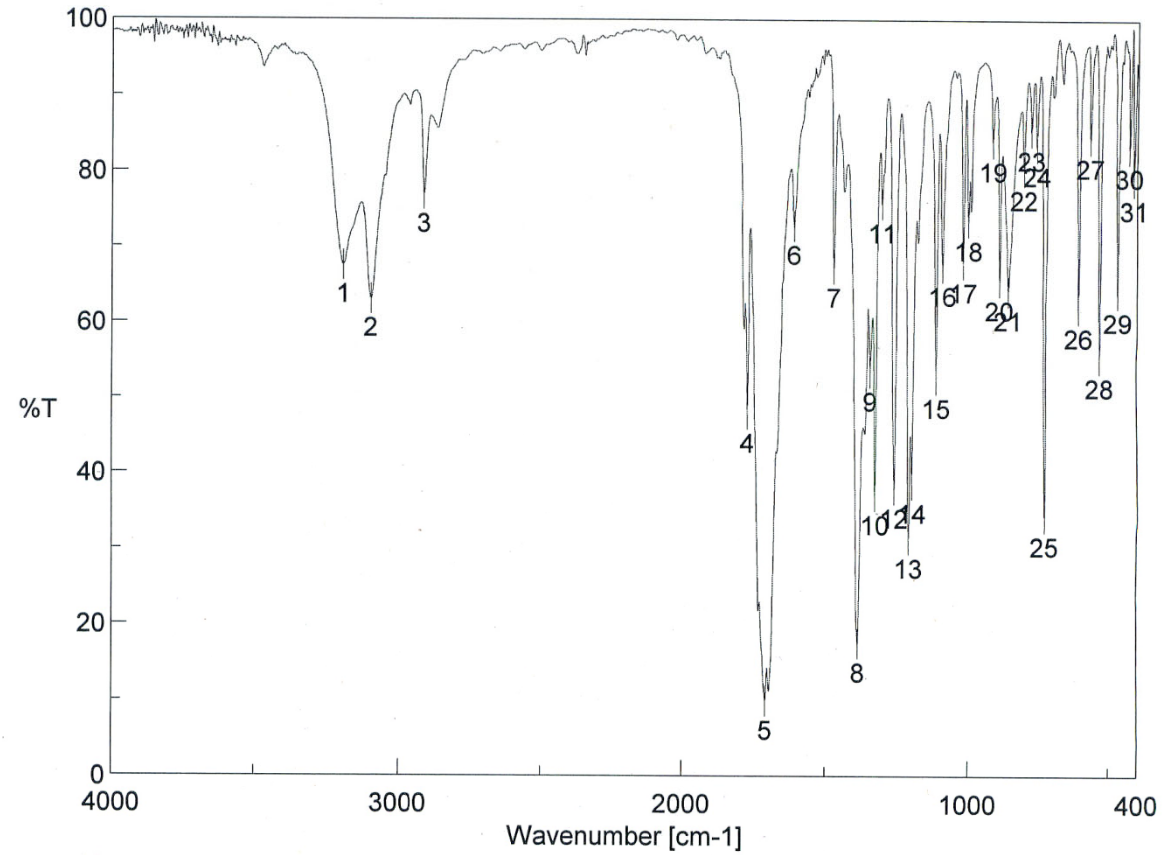

ESI MS spectra of (±)-**1**

calculated for $C_{13}H_{10}N_2NaO_4$ ($[M + Na]^+$) 281.05, found 280.90

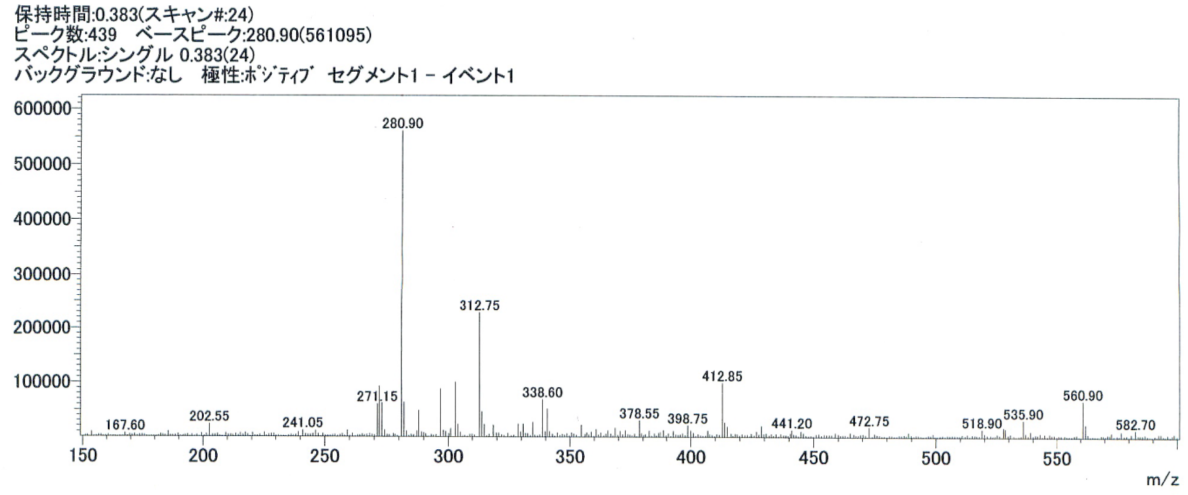

IR (KBr) spectra of (±)-**2**

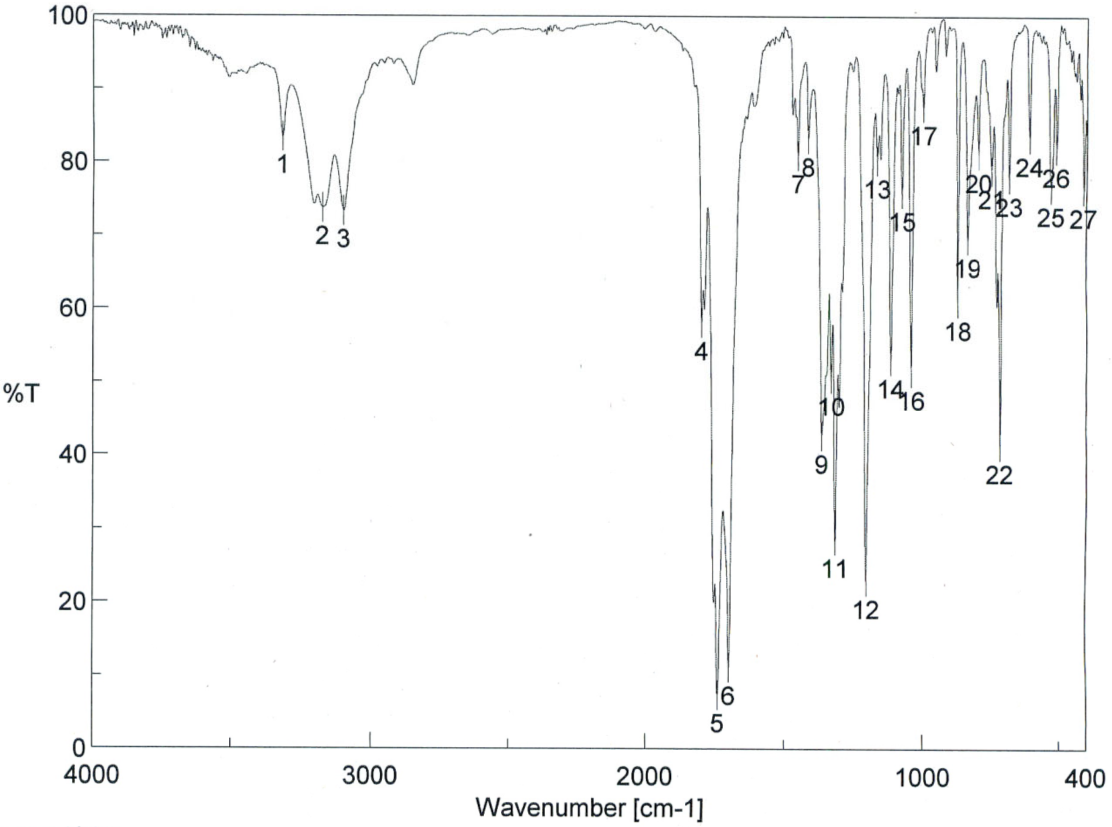

ESI MS spectra of (±)-**2**

calculated for $C_{13}H_9FN_2NaO_4$ ($[M + Na]^+$) 299.04, found 298.95

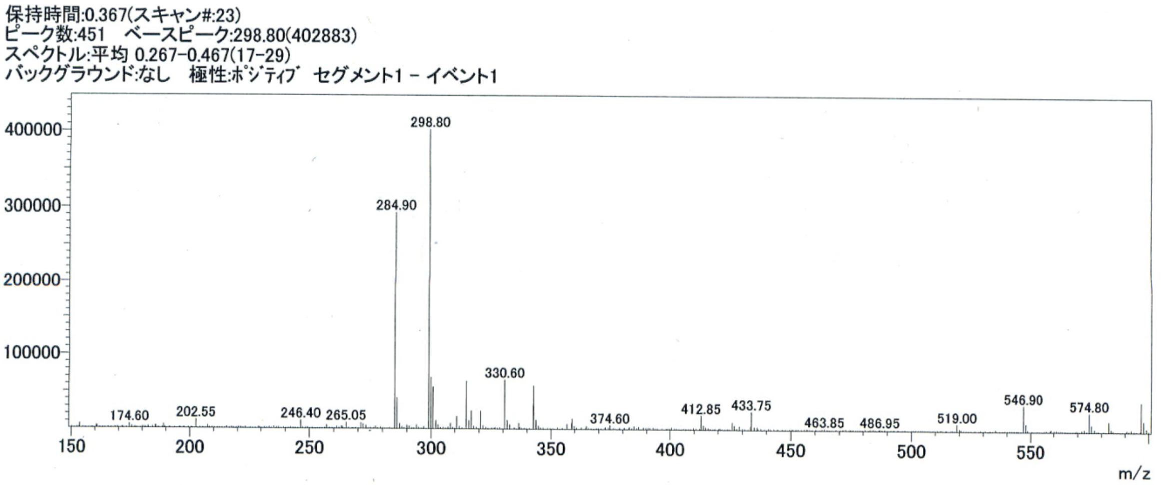

Supplement: Supplementary file 1 [file SC-006-C4SC03047H-s001.pdf]
